# SIGMA PHI OMEGA CHAPTERS AND THEIR COLLABORATION WITH THE PILOTED GSA STUDENT CHAPTERS

**DOI:** 10.1093/geroni/igac059.184

**Published:** 2022-12-20

**Authors:** Marilyn Gugliucci

**Affiliations:** University of New England College of Osteopathic Medicine, Biddeford, Maine, United States

## Abstract

Networking is the action or process of interacting with others to exchange information and develop professional or social contacts. The newly piloted GSA student chapters are encouraged to build relationships that can lead to networking and collaboration. While it is important to maintain a unique Sigma Phi Omega institutional chapter identity, the GSA student chapters also focus on the field of aging within a Higher Education Institution. Sigma Phi Omega collaborating with GSA Student Chapters is mutually beneficial in expanding learning, exploring new ideas, building professional connections, creating innovative projects, and gaining insights to other opportunities for professional and personal growth. This session will provide guidance on how to foster collaboration and growth for each of these chapters while honoring respective chapter missions, requirements, and educational experiences.

